# “Internet Addiction”: a Conceptual Minefield

**DOI:** 10.1007/s11469-017-9811-6

**Published:** 2017-09-19

**Authors:** Francesca C. Ryding, Linda K. Kaye

**Affiliations:** Department of Psychology, Edge Hill University, St Helens Road, Ormskirk, Lancashire L39 4QP UK

**Keywords:** Internet addiction, Gratifications, Contexts, Platforms, IGD

## Abstract

With Internet connectivity and technological advancement increasing dramatically in recent years, “Internet addiction” (IA) is emerging as a global concern. However, the use of the term ‘addiction’ has been considered controversial, with debate surfacing as to whether IA merits classification as a psychiatric disorder as its own entity, or whether IA occurs in relation to specific online activities through manifestation of other underlying disorders. Additionally, the changing landscape of Internet mobility and the contextual variations Internet access can hold has further implications towards its conceptualisation and measurement. Without official recognition and agreement on the concept of IA, this can lead to difficulties in efficacy of diagnosis and treatment. This paper therefore provides a critical commentary on the numerous issues of the concept of “Internet addiction”, with implications for the efficacy of its measurement and diagnosticity.

## What Is Internet Addiction (IA)?

Traditionally, the term addiction has been associated with psychoactive substances such as alcohol and tobacco; however, behaviours including the use of the Internet have more recently been identified as being addictive (Sim et al. 2012). The concept of IA is generally characterised as an impulse disorder by which an individual experiences intense preoccupation with using the Internet, difficulty managing time on the Internet, becoming irritated if disturbed whilst online, and decreased social interaction in the real world (Tikhonov and Bogoslovskii 2015). These features were initially proposed by Young (1998) based on the criteria for pathological gambling (Yellowlees and Marks 2007), and have since been adapted for consideration within the DSM-5. This has been well received by many working in the field of addiction (Király et al. 2015; Petry et al. 2014), and has been suggested to enable a degree of standardisation in the assessment and identification of IA (King and Delfabbro 2014). However, there is still debate and controversy surrounding this concept, in which researchers acknowledge much conceptual disparity and the need for further work to fully understand IA and its constituent disorders (Griffiths et al. 2014).

Much of the debate relates to the issue that IA is conceptualised as addiction to the Internet as a singular entity, although it incorporates an array of potential activities (Van Rooij and Prause 2014). That is, the Internet, in all its formats, whether accessed via PC, console, laptop or mobile device, is fundamentally a portal through which we access activities and services. Internet connectivity thus provides us with ways of accessing the following types of activities; play (e.g. online forms of gaming, gambling), work (accessing online resources, downloading software, emailing, website hosting), socialising (social networking sites, group chats, online dating), entertainment (film databases, porn, music), consumables (groceries, clothes), as well as many other activities and services. In this way, the Internet is a highly multidimensional and diverse environment which affords a multitude of experiences as a product of the specific virtual domain. Thus, it is questionable as to whether there is any degree of consistency in the concept of IA, in light of these diverse and specific affordances which may relate to Internet engagement. Indeed, it has been indicated that there are several distinct types of IA, including online gaming, social media, and online shopping (Kuss et al. 2013), and it has been claimed that through engagement in these behaviours, individuals may become addicted to these experiences, as opposed to the medium itself (Widyanto et al. 2011). Thus, IA is arguably too generalised as a concept to adequately capture these nuances. That is, an individual who spends excessive time online for shopping is qualitatively different from someone who watches or downloads porn excessively. These represent distinct behaviours which are arguably underpinned by different gratifications. Thus, the functionality of aspects of the Internet is a key consideration for research in this area (Tokunaga 2016). This is perhaps best approached from a uses and gratifications perspective (LaRose et al. 2003; Larose et al. 2001a; Wegmann et al. 2015), to more fully understand the aetiology of IA (discussed subsequently). This is often best underpinned by the uses and gratifications theory (Larose et al. 2001a, 2003), which seeks to explain (media) behaviours by understanding their specific functions and how they gratify certain needs. Indeed, in the context of IA, this may be particularly useful to establish the extent to which certain Internet-based behaviours may be more or less functional in need gratification than others, and the extent to which it is Internet platform itself which is driving usage or indeed the constituent domains which it affords. If the former, then controlling Internet-based usage behaviour more generically is perhaps appropriate, however, a more specified approach may often be required given the diverse needs the online environment can afford users.

## IA from a Gratifications Perspective

It is questionable on the extent to which IA is itself the “addiction” or whether its aetiology relates to other pre-existing conditions, which may be gratified through Internet domains (Caplan 2002). One particular theory that has been referenced throughout much developing research (King et al. 2012; Laier and Brand 2014) is the cognitive-behavioural model, proposed by Davis (2001). This model suggests that maladaptive cognitions precede the behavioural symptoms of IA (Davis 2001; Taymur et al. 2016). Since much research focuses on the comorbidity between IA and psychopathology (Orsal et al. 2013), this is particularly useful in underpinning the concept of IA, and perhaps provides support that IA is a manifestation of underlying disorders, due to its psychopathological aetiology (Taymur et al. 2016). Additionally, the cognitive-behavioural model also distinguishes between both specific and generalised pathological Internet use, in comparison to global Internet behaviours that would not otherwise exist outside of the Internet, such as surfing the web (Shaw and Black 2008). As such, it would assume those individuals who spend excessive time playing poker online, for example, are perhaps better categorised as problematic gamblers rather than as Internet addicts (Griffiths 1996). This has been particularly advantageous in the contribution to defining IA, as earlier literature tended to focus solely on either content-specific IA, or the amount of time spent online, rather than focussing as to why individuals are actually online (Caplan 2002). Indeed, this shows promise in resolving some of the aforementioned issues in the specificity of IA, as well as the likelihood of pre-existing conditions underpinning problematic behaviours on the Internet.

Much of the recent literature in the realm of IA has focused upon Internet Gaming Disorder (IGD) which has recently been included as an appendix as “a condition for further study” in the DSM-5 (American Psychiatric Association 2013). This has driven a wide range of research which has sought to establish the validity of IGD as an independent clinical condition (Kuss et al. 2017). Among the wealth of research papers surrounding this phenomenon, there remains large disparity within the academic community. Although some researchers claim there is consensus on IGD as a valid clinical disorder (Petry et al. 2014), others do not support this (e.g. Griffiths et al. 2016). As such, the academic literature has some way to go before more established claims can be made towards IGD as a valid construct, and indeed how this impacts upon clinical treatment.

One means by which researchers could move forward in this regard is to establish the validity of IGD to a wider range of gaming formats. That is, IGD research has predominantly defined the reference point in studies as “online games” or in some cases, is has been even less specific (Lemmens et al. 2015; Rehbein et al. 2015; Thomas and Martin 2010). Arguably, there are a range of forms of “online” gaming, including social networking site (SNS) games which are Internet-mediated and thus by definition, would appear under the remit of IGD. Indeed, links between SNS and gaming have been previously noted (Kuss and Griffiths 2017), although this has not specifically been empirically explored in the context of IGD symptomology. For example, causal form of gaming as is typically the case for SNS gaming have their own affordances in respect of where and how they are played, given these are often played on mobile devices rather than on more traditional PC or console platforms. Further, the demographics of who are most likely to play these games can vary from others forms of gaming which have predominated the IGD literature (Hull et al. 2013; Leaver and Wilson 2016). Accordingly, these affordances present additional nuances, which the literature has not yet fully accounted for in its exploration of IGD. Clearly, IGD relates to a specific form of Internet behaviour which may be conceptualised within IA, yet is paramount to understand it as a separate entity to ensure the conceptualisation and any associated treatment provision is sufficiently nuanced. Likewise, the same case can be made for many other Internet-based behaviours which may be best being established in respect of their functionality and gratification purposes for users.

## IA as a Contextual Phenomenon

There is growing evidence suggesting that context is key towards the processes and cognitions associated with consumption of substances such as alcohol (Monk and Heim 2013, 2014; Monk et al. 2016), highlighting some important implications towards understanding IA, as a form of behavioural addiction. That is, the study of IA has rarely been studied in respect of its contextual affordances, even though the combination of Internet connectivity (WiFi) and mobility (smartphones) means that the Internet may be accessed in many ways and in multiple contexts. It has been indicated by Griffiths (2000) that few studies consider the context of Internet use, despite many users spending a substantial amount of time on the Internet via the use of different platforms, such as mobile devices, as opposed to a computer (Hadlington 2015). It has been highlighted by Kawabe et al. (2016) that smartphone ownership in particular is rapidly increasing, and for some, smartphone devices have become a substitute for the computer (Aljomaa et al. 2016). It has also been suggested that the duration of usage on smartphones have been significantly associated with IA (Kawabe et al. 2016). This can largely be attributed to the advancement of smartphone technology, which permit them to function as a “one-stop-shop” for a variety of our everyday needs (checking the time, replying to emails, listening to music, interacting with others, playing games), and thus it is understandable that we are spending more of our time in using these devices. This further implicates research in IA, as this has often focussed on users’ Internet engagement through computers as opposed to mobile devices, albeit the numerous Internet subtypes accessible through mobile devices (Sinkkonen et al. 2014). One Internet subtype in particular which may facilitate addictive behaviours are social networking sites such as Facebook (Wu et al. 2013). Particularly, research has identified a positive relationship between daily usage of smartphones and addictive symptoms towards Facebook (Wu et al. 2013). This may also be the case for behaviours such as gaming through SNS which are typically accessed on mobile devices rather than computers. However, of critical interest here, is that addiction to these games has been argued to fall under the classification of IGD, despite being online via Facebook (Ryan et al. 2014). This indicates that the platform of Internet access is important in online behaviours, as well as implicating that further distinction between Internet subtypes should be made (particularly within SNS), to establish the different features of these, and how these affordances may be related to excessive usage. This issue is particularly pertinent given the increased interest in “smartphone addiction” (Kwon et al. 2013) in which the name assumes we are simply studying addiction to our smartphones themselves, not necessarily the functions they are affording to us. Research such as this is assuming the “problem” is the interaction with the technology (e.g. specific device) itself, when this is most likely not the case. Indeed, recent evidence highlights that different uses/functions of smartphones may be more likely to prompt users to feel more “attached” to the device than others, and that usage is often framed by one’s current context (Fullwood et al. 2017).

In addition to being able to access the Internet through multiple platforms, we are often reliant on the Internet for many everyday tasks, which poses a further issue in conceptualising what is “problematic” compared to “required” usage. The increased exposure to the Internet in both work and education make it difficult to avoid usage in such environments (Kiliҫer and Ҫoklar 2015; Uçak 2007), and it could be argued that the amount of time spent on the Internet for such contexts cannot be reflected as an addiction (LaRose et al. 2003). This is pertinent in light of much research, which tends to rely on metrics such as time spent online (e.g. average hours per week) as a variable in research paradigms. Particularly, this tends to be used to correlate against other psychological factors, such as depression or well-being, to indicate how “internet use” may be a problematic predictor of these outcomes (e.g. Sanders et al. 2000). In light of the aforementioned issues, this does not offer any degree of specificity in how time spent online is theoretically related to the outcomes variables of interest (Kardefelt-Winther 2014). Other studies have approached this with greater nuance by considering specific activities, such as number of emails sent and received in a given time period (Ford and Ford 2009; LaRose et al. 2001b), or studied Internet use for a variety of different purposes, such as for health purposes and communication (Bessière et al. 2010). Further, other researchers have highlighted the distinction between behaviours such as smartphone “usage” versus “checking” (Andrews et al. 2015), whereby the latter may represent a more compulsive and less consciously driven and potentially more addictive form of behaviour than actual “usage”. These more nuanced approaches provide a more useful and theoretically insightful means of establishing how time spent online may be psychologically relevant as a concept. This suggests that future research which theorises on the impacts of “time spent online” (or “screen-time use”) should provide distinction between usage for work/education and leisure, and the gratification this engagement affords, to obtain greater nuance beyond the typical flawed metrics such as general time spent online.

A further compliment to the existing IA literature would be greater use of behavioural measures which garner users’ actual Internet-based behaviours. This is particularly relevant when considering that almost all existing research on smartphone addiction or problematic use, for example has been based on users’ self-reported usage, with no psychometric measure being validated against behavioural metrics. Worryingly, it has been noted that smartphone users grossly underestimate the amount of times they check their smartphone on a daily basis, with digital traces of their smartphone behaviours illuminating largely disparate findings (Andrews et al. 2015). Clearly, there is much opportunity to establish forms of Internet usage by capitalising on behavioural metrics and digital traces rather than relying on self-report which may not always be entirely accurate.

## Conclusion

The concept of IA is more complex than it often theorised. Although there have been multiple attempts to define the characteristics of IA, there a numerous factors which require greater clarity in the theoretical underpinnings of this concept. Specifically, IA is often considered from the perspective that the Internet itself (and indeed the technology through which we access it) is harmful, with little specificity in how this functions in different ways for individual users, as well as the varying affordances which can be gained through it. Unfortunately, this aligns somewhat with typical societal conceptions of “technology is harmful” perspective, rather than considering the technology itself is simply a portal through which a psychological need is being served. This perspective is not a new phenomenon. Most new media has been subject to such moral panic and thus this serves a historical tradition within societal conception of new media. Indeed, this has been particularly relevant to violent videogames which scholars have discussed in respect of this issue (Ferguson 2008). Whilst many scholars recognise this notion through the application of a user and gratifications perspective, stereotypical conceptions of “technology is harmful” still remain. This raises the question about how we as psychologists can enable a cultural shift in these conceptions, to provide a more critical perspective on such issues. The pertinence of this surrounds two key issues; firstly that moving beyond a “technology is harmful” perspective, particularly for concerns over “Internet addiction” as one example, can enable a more critical insight into the antecedents of problematic behaviour to aid treatment, rather than simply revoking access from the Internet for such individuals. Arguably, this latter strategy would not always address the route of the issue and raises implications about the extent to which recidivism would occur upon reinstating Internet access. Secondly, on a more general level, diverging from an “anti-technology” perspective can enable researchers to draw out the nuances of specific Internet environments and their psychological impacts rather than battling with more blanket assumptions that “technology” (as a unitary concept) is presenting all individuals with the same issues and affordances, regardless of the specific virtual platform or context. In this way, we may be presented with more plentiful opportunities to more critically explore individuals and their interactions across many Internet-mediated domains and contexts.

## References

[CR1] Aljomaa SS, Al Qudah MF, Albursan IS, Bakhiet SF, Abduljabbar AS (2016). Smartphone addiction among university students in the light of some variables. Computers in Human Behavior.

[CR2] American Psychiatric Association (2013). Diagnostic and statistical manual of mental disorders.

[CR3] Andrews S, Ellis DA, Shaw H, Piwek L (2015). Beyond self-report: tools to compare estimated and real-world smartphone use. PloS One.

[CR4] Bessière K, Pressman S, Kiesler S, Kraut R (2010). Effects of Internet use on health and depression: a longitudinal study. Journal of Medical Internet Research.

[CR5] Caplan SE (2002). Problematic Internet use and psychosocial well-being: development of a theory-based cognitive–behavioral measurement instrument. Computers in Human Behavior.

[CR6] Davis RA (2001). A cognitive-behavioral model of pathological Internet use. Computers in Human Behavior.

[CR7] Ferguson CJ (2008). The school shooting/violent video game link: casual relationship or moral panic?. Journal of Investigative Psychology and Offender Profiling.

[CR8] Ford, G. S., & Ford, S. G. (2009). *Internet use and depression among the elderly*. Phoenix Center Policy Paper Series. Retrieved April 26, 2016, from http://www.phoenix-center.org/pcpp/PCPP38Final.pdf.

[CR9] Fullwood C, Quinn S, Kaye LK, Redding C (2017). My Virtual friend: a qualitative analysis of the attitudes and experiences of Smartphone users: implications for Smartphone attachment. Computers in Human Behavior.

[CR10] Griffiths MD (1996). Gambling on the Internet: a brief note. Journal of Gambling Studies.

[CR11] Griffiths M (2000). Internet addiction - time to be taken seriously?. Addiction Research & Theory.

[CR12] Griffiths MD, King DL, Demetrovics Z (2014). DSM-5 Internet Gaming Disorder needs a unified approach to assessment. Neuropsychiatry.

[CR13] Griffiths MD, Van Rooij AJ, Kardefelt-Winther D (2016). Working towards an international consensus on criteria for assessing internet gaming disorder: a critical commentary on Petry et al. (2014). Addiction.

[CR14] Hadlington LJ (2015). Cognitive failures in daily life: exploring the link with Internet addiction and problematic mobile phone use. Computers in Human Behavior.

[CR15] Hull DC, Williams GA, Griffiths MD (2013). Video game characteristics, happiness and flow as predictors of addiction among video game players: a pilot study. Journal of Behavioral Addictions.

[CR16] Kardefelt-Winther D (2014). A conceptual and methodological critique of internet addiction research: towards a model of compensatory internet use. Computers in Human Behavior.

[CR17] Kawabe K, Horiuchi F, Ochi M, Oka Y, Ueno S (2016). Internet addiction: prevalence and relation with mental states in adolescents. Psychiatry and Clinical Neurosciences.

[CR18] Kiliҫer K, Ҫoklar AN (2015). Examining human value development of children with different habits of Internet usage. Hacettepe University of Education.

[CR19] King DL, Delfabbro PH (2014). The cognitive psychology of Internet gaming disorder. Clinical Psychology Review.

[CR20] King DL, Delfabbro PH, Griffiths MD, Gradisar M (2012). Cognitive-behavioral approaches to outpatient treatment of Internet addiction in children and adolescents. Journal of Clinical Psychology.

[CR21] Király O, Griffiths MD, Demetrovics Z (2015). Internet gaming disorder and the DSM-5: conceptualization, debates, and controversies. Current Addiction Reports.

[CR22] Kuss DJ, Griffiths MD (2017). Social networking networking sites and addiction: ten lessons learned. International Journal of Environmental Research and Public Health.

[CR23] Kuss DJ, van Rooij AJ, Shorter GW, Griffiths MD, van de Mheen D (2013). Internet addiction in adolescents: prevalence and risk factors. Computers in Human Behavior.

[CR24] Kuss, D. J., Grittihs, M. D., & Pontes, H. M. (2017). DSM-5 diagnosis of Internet Gaming Disorder: some ways forward in overcoming issues and concerns in the gaming studies field. *Journal of Behavioral Addictions.*10.1556/2006.6.2017.032. 10.1556/2006.6.2017.032 PMC5520128 28662619

[CR25] Kwon M, Lee J, Won W, Park J, Min J, Hahn C, Gu X, Choi J, Kim D (2013). Development and validation of a smartphone addiction scale. PloS One.

[CR26] Laier C, Brand M (2014). Empirical evidence and theoretical considerations on factors contributing to cybersex addiction from a cognitive-behavioral view. Sexual Addiction & Compulsivity.

[CR27] Larose R, Mastro D, Eastin MS (2001). Understanding Internet usage: a social-cognitive approach to uses and gratifications. Social Science Computer Review.

[CR28] LaRose, R., Eastin, M. S., & Gregg, J. (2001b). Reformulating the Internet paradox: social cognitive explanations of Internet use and depression. *Journal of Online Behavior, 1*(2). Retrieved Setpember 12, 2017 from http://psycnet.apa.org/record/2002-14047-001.

[CR29] LaRose R, Lin CA, Eastin MS (2003). Unregulated Internet usage: Addiction, habit, or deficient self-regulation?. Media Psychology.

[CR30] Leaver T, Wilson M (2016). Social networks, casual games and mobile devices: the shifting contexts of gamers and gaming.

[CR31] Lemmens JS, Valkenburg PM, Gentile DA (2015). The internet gaming disorder scale. Psychological Assessment.

[CR32] Monk RL, Heim D (2013). Environmental context effects on alcohol-related outcome expectancies, efficacy and norms: a field study. Psychology of Addictive Behaviors.

[CR33] Monk RL, Heim D (2014). A real-time examination of context effects on alcohol cognitions. Alcoholism: Clinical and Experimental Research.

[CR34] Monk RL, Pennington CR, Campbell C, Price A, Heim D (2016). Implicit alcohol-related expectancies and the effect of context. Journal of Studies on Alcohol and Drugs.

[CR35] Orsal O, Unsal A, Ozalp SS (2013). Evaluation of Internet addiction and depression among university students. Procedia - Social and Behavioral Sciences.

[CR36] Petry NM, Rehbein F, Gentile DA, Lemmens JS, Rumpf H, Mӧßle T (2014). An international consensus for assessing Internet Gaming Disorder using the new DSM-5 approach. Addiction.

[CR37] Rehbein F, Kliem S, Baier D, Mossle T, Petry NM (2015). Prevalence of internet gaming disorder in German adolescents: diagnostic contribution of the nine DSM-5 criteria in a state-wide representative sample. Addiction.

[CR38] Ryan T, Chester A, Reece J, Xenos S (2014). The uses and abuses of Facebook: a review of Facebook addiction. Journal of Behavioral Addictions.

[CR39] Sanders CE, Field TM, Diego M, Kaplan M (2000). The relationship between Internet use to depression and social isolation among adolescents. Adolescence.

[CR40] Shaw M, Black DW (2008). Internet addiction. CNS Drugs.

[CR41] Sim T, Gentile DA, Bricolo F, Serpelloni G, Gulamoydeen F (2012). A conceptual review of research on the pathological use of computers, video games, and the Internet. International Journal of Mental Health and Addiction.

[CR42] Sinkkonen H-M, Puhakka H, Meriläinen M (2014). Internet use and addiction among Finnish adolescents (15–19 years). Journal of Adolescence.

[CR43] Taymur I, Budak E, Demirci H, Akdağ HA, Güngör BB, Özdel K (2016). A study of the relationship between internet addiction, psychopathology and dysfunctional beliefs. Computers in Human Behavior.

[CR44] Thomas NJ, Martin FH (2010). Video-arcade game, computer game and Internet activities of Australian students: participation participation habits and prevalence of addiction. Australian Journal of Psychology.

[CR45] Tikhonov MN, Bogoslovskii MM (2015). Internet addiction factors. Automatic Documentation and Mathematical Linguistics.

[CR46] Tokunaga RS (2016). An examination of functional difficulties from Internet use: Media habit and displacement theory explanations. Human Communication Research.

[CR47] Uçak NÖ (2007). Internet use habits of students of the department of information management, Hacettepe University, Ankara. The Journal of Academic Librarianship.

[CR48] Van Rooij A, Prause N (2014). A critical review of “Internet addiction” criteria with suggestions for the future. Journal of Behavioral Addictions.

[CR49] Wegmann E, Stodt B, Brand M (2015). Addictive use of social networking sites can be explained by the interaction of Internet use expectancies, Internet literacy, and psychopathological symptoms. Journal of Behavioral Addictions.

[CR50] Widyanto L, Griffiths MD, Brunsden V (2011). A psychometric comparison of the Internet Addiction Test, the Internet-Related Problem Scale, and self-diagnosis. Cyberpsychology, Behavior, and Social Networking.

[CR51] Wu AMS, Cheung VI, Ku L, Hung EPW (2013). Psychological risk factors of addiction to social networking sites among Chinese smartphone users. Journal of Behavioral Addictions.

[CR52] Yellowlees PM, Marks S (2007). Problematic Internet use or Internet addiction?. Computers in Human Behavior.

[CR53] Young KS (1998). Internet addiction: the emergence of a new clinical disorder. Cyberpsychology & Behavior.

